# Colorectal cancer molecular profiling: Opportunities for early detection

**DOI:** 10.1002/ctm2.70474

**Published:** 2025-09-29

**Authors:** Alexandra Sala, Lisa van den Driest, Nicholas J. W. Rattray, Caroline H. Johnson, James M. Cameron, Sajid A. Khan, David S. Palmer, Matthew J. Baker

**Affiliations:** ^1^ Dxcover Ltd Glasgow UK; ^2^ Strathclyde Institute of Pharmacy and Biomedical Sciences (SIPBS) University of Strathclyde Glasgow UK; ^3^ Yale School of Public Health Yale University New Haven Connecticut USA; ^4^ Yale School of Medicine Yale University New Haven Connecticut USA; ^5^ Department of Pure and Applied Chemistry University of Strathclyde Glasgow UK; ^6^ School of Medicine Faculty of Clinical and Biomedical Sciences University of Lancashire Preston UK

**Keywords:** cancer, colorectal, CRC, detection, diagnostics, early‐onset, early‐stage, liquid biopsy

## Abstract

Colorectal cancer (CRC) is one of the most common and deadliest cancers worldwide, and incidence rates are rising. However, early detection and intervention can improve the survival rates and quality of life of affected patients. Current screening tests used to streamline patients into colonoscopy either lack test adherence or sensitivity for detecting premalignant and early‐stage CRC, reducing the advantages of screening measures. Cost‐effective and minimally invasive diagnostic tests which can detect immune system and metabolic changes are key to lower the incidence of CRC advanced stages. We herein discuss the statistics, risk factors and unique genetic characteristics of CRC, focussing on the importance of understanding non tumour‐derived information in premalignant states for developing comprehensive techniques to achieve earlier diagnosis of CRC. Moreover, the advantages and limitations of current UK and USA screening programmes and emerging detection tools are discussed, along with prospective diagnostics such as genomics, proteomics and spectroscopy.

## Graphical abstract

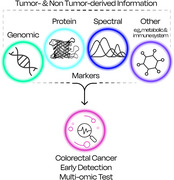

## 1 EARLIER DETECTION OF CRC IS NEEDED: WHY NOW AND HOW?

Colorectal cancer (CRC) incidence has been predicted to rise steadily in the next two decades, with cases among young people already on the rise.^1^, ^2^, ^3^ Estimates show increased mortality rates worldwide and across all age groups.^3^, ^4^, ^5^, ^6^, ^7^ The average 5‐year survival rate after diagnosis decreases from 91% in early‐stage CRC, to as low as 15% for stage IV CRC;^8^ early detection and intervention can improve the survival rates and quality of life of patients. Average‐risk screening has the potential to prevent CRC and reduce CRC‐related morbidity and mortality. The tumorigenesis of CRC is a complex process, whereby pre‐cancerous lesions, such as advanced adenomas, transform into cancer over several months or years. This suggests there should be a larger window for potential intervention, but screening inefficiencies, such as low adherence and limited test performance, means that many CRC patients are still often diagnosed with late‐stage disease. Due to the lack of available resources, the invasiveness of colonoscopy, and the relatively low sensitivity (and specificity) of stool‐based tests, there is a need for alternative screening strategies.

Worldwide, colonoscopy is still considered the gold standard technique for the diagnosis of CRC. However, in many international healthcare systems, it is often prioritized for high risk patients, with suspected gastro‐intestinal symptoms or positive screening results.^9^, ^10^ Unfortunately, symptoms may only appear in later stages of tumorigenesis, meaning many early‐stage CRC are diagnosed when the cancer is more advanced. Evaluating alternative diagnostic and/or screening tools that are less invasive, more accurate and cost‐effective, could streamline people in further confirmatory testing (i.e., colonoscopy). More efficient screening options could reduce colonoscopy waiting times and pave the way for early detection of CRC.

In this review article, we discuss the statistics, risk factors and genetic characteristics of CRC. Moreover, we assess the current state of CRC diagnostic pathways in UK and USA. Emerging screening and diagnostic tools are also discussed, along with their advantages and limitations.

## 2 COLORECTAL CANCER: DEMOGRAPHICS AND STATISTICS

CRC is the third most common cancer worldwide, accounting for around 2 million new cases every year and representing over 10% of all new cancer cases per year.^11^ 90% of new cases are registered in older individuals (i.e., late‐onset; people aged 50 and above);^11^ however, early‐onset (i.e., people aged 49 and below) of CRC is predicted to increase by over 15% in the next 20 years, with rates higher in males and low‐medium human development index (HDI) regions.^3^ CRC cases are expected to rise in several countries, such as the UK (+3%) and USA (+17%).^3^ This is still one of the most fatal cancers worldwide, with approximately 1 million deaths every year, second only to lung cancer.^11^

## 3 TUMORIGENESIS: FROM ADENOMA TO MALIGNANT GROWTH

The development of CRC begins with the acquisition of mutations that initiate the formation of benign adenomatous polyps. Polyps are growths that originate from mucous membranes, and can present characteristic of an adenoma, a benign tumour that is prone to become cancerous.^12^ Adenomatous polyps can reside in the colon for years before spontaneously transitioning into malignancy. While most somatic mutations are neutral and do not influence cell growth and/or survival, a subset of mutations, known as driver mutations, provide a clonal growth advantage during tumorigenesis.

A primary driver mutation known to initiate the transition from healthy cell into adenoma is the inactivation of the adenomatous polyposis coli (APC) tumour suppressor gene, found in about 80% of CRC.^13^, ^14^ The APC gene is crucial in the Wnt signalling pathway, primarily through its role in regulating the degradation of β‐catenin. β‐catenin functions as a transcription factor that activates genes involved in cell proliferation, initiating the formation of adenomatous polyps. For the development into carcinomas, an APC mutation must be followed by consecutive deleterious mutations.^15^

**FIGURE 1:**
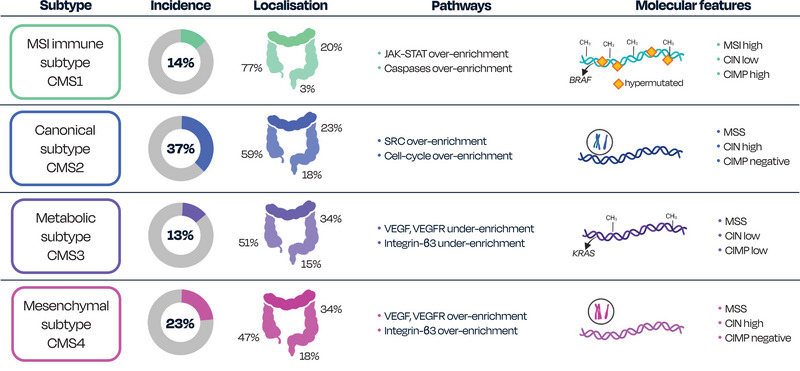
Overview of consensus molecular subtypes (CMS) in colorectal cancer. The figure specifies differences in incidence, localization, pathways enrichment and molecular features among the four CMS subtypes. CMS1 is characterized by high MSI and CIMP, with a high incidence of BRAF mutations. CMS2 is defined by high CIN and overexpression of Wnt and MYC pathways. CMS3 is characterized by metabolic dysregulation and KRAS mutations. CMS4 also displays high CIN, as well as over‐enrichment of VEGF/VEGFR and Integrin‐β3 pathways. Additional distinctions include differences in methylation and mutation statuses across subtypes. [BRAF, B‐Raf proto‐oncogene; CMS, consensus molecular subtypes; CIMP, CpG island methylator phenotype; CIN, chromosomal instability; JAK, Janus kinase; KRAS, KRAS proto‐oncogene; MSI, microsatellite instability; STAT, signal transducer and activator of transcription; SRC, SRC proto‐oncogene; MSS, microsatellite stable; VEGF, vascular endothelial growth factor; VEGFR, vascular endothelial growth factor receptor].

The transition from adenoma to carcinoma is often accompanied by the acquisition of additional mutations in tumour suppressor genes such as KRAS, TP53, or SMAD2 and SMAD4 genes of the transforming growth factor β (TGF‐ β) pathway. In addition to driver mutations, at least three major molecular pathways have been identified; the most common (70%), is chromosomal instability (CIN) pathway, characterized by the accumulation of chromosomal abnormalities.^13^, ^16^ Another pathway involves microsatellite instability (MSI) caused by dysfunctional mismatch repair (MMR) genes, leading to hypermutability. In addition, CpG Island methylator phenotype (CIMP) represents an epigenetic phenomenon resulting in hypermethylation of the gene promoter for the MMR enzyme, leading to gene silencing. These characteristics can help stratify CRC patients into four gene expression consensus molecular subtypes (CMS): CMS1 (immune), CMS2 (canonical), CMS3 (metabolic), and CMS4 (mesenchymal).^13^, ^17^ These subtypes differ in incidence, localization, and molecular features as described in Figure 1. CRC presents as highly heterogeneous at both genomic and transcriptomic levels and it is still controversial whether gene expression signatures can contribute to the clinically relevant picture of the four existing CMS.^17^, ^18^

### 3.1 The early‐onset case

Early‐onset of CRC is a phenomenon that has been gaining interest in the past few decades and is discussed in several reviews.^2^, ^4^, ^5^, ^19^, ^20^ Siegel *et al.* highlighted that in the USA, deaths from CRC in men aged 20 to 39 surpassed those from brain tumours in 2021.^1^ In addition, CRC was the primary cause of cancer‐related death in men aged 40 to 49. In women aged 40 to 49, CRC was the second leading cause of cancer‐related death in 2021, second only to breast cancer.^1^

Statistical research conducted over a decade ago estimated that CRC diagnosis in individuals aged younger than 50 would increase steadily by approximately 10–12% by 2030.^4^, ^5^, ^6^ However, in 2018, it was reported that early‐onset CRC already accounts for approximately 10% of all new diagnosis of CRC.^4^, ^21^ Currently, there are no specific diagnostic tests for early‐onset CRC. To counteract the alarming rise in cases, the U.S. Preventive Services Task Force and the American Cancer Society lowered the minimum age for recommended screening, allowing people aged 45 to 49 to partake in the national screening programme.^22^

A birth cohort effect (i.e., variation in the risk of a health outcome based on the birth year) has been linked to the increasing incidence of early‐onset CRC in individuals aged 50 to 54, and the flattening of a previously decreasing incidence in those aged 55 to 74, which are due to shared exposures among people born after 1960.^23^ Some of the risk factors, which show a consistent pattern of increased association with early‐onset, include those that have been reported previously for CRC at all ages; being obese or overweight, and excessive alcohol consumption.^24^, ^25^

Dietary patterns, such as the Western diet which includes “fast foods” (i.e., low fibre, high fat and ultra‐processed foods), and individual dietary constituents such as sugar‐sweetened beverages and processed meat, as well as diet‐associated disparities such as food insecurity, have all been associated with increased risk of early‐onset in isolated studies.^23^, ^26^ Other factors that have been linked with early‐onset include environmental exposures, and dysbiosis of the gut microbiota caused by antibiotic exposure, alcohol consumption and various components of the diet.^20^ However, it is probable that the increased rates of early‐onset CRC are due to a mixture of these risk factors that are more prevalent and at higher doses in those born after 1960, along with an interplay of these exposures on a particular genetic background. Therefore, the birth cohort effect is an important consideration in which exposures and risk factors could be pinpointed to help understand the aetiology of increasing rates of early‐onset CRC.

It is still unclear whether early‐onset CRC completely differs from CRC (i.e., late‐onset). Nonetheless, evidence suggests variance in clinical and pathological features, invasive behaviour and molecular profiles. Early‐onset CRC often present at a more aggressive and advanced stage (i.e., III‐IV) at diagnosis than late‐onset CRC, resulting in a greater impact on life for younger patients.^4^ This suggests that there may be differences in tumour biology; 30% of early‐onset cases are related to diverse hereditary cancer syndromes, including familial adenomatous polyposis (FAP) and MUTYH associated polyposis (MAP).^5^ Early‐onset CRC is also associated with a higher occurrence of multiple CRCs within and after 6 months from the first diagnosis.^19^ Further studies still need to shed light on this increasing phenomenon, currently investigated by several researchers worldwide.^27^, ^28^

### 3.2 The unmet need of early detection

Early detection can allow patients to receive timely surgical resection and therapeutics, positively impacting overall outcomes. There are various methods for detecting CRC; including colonoscopy, imaging, tissue biopsy, and biomarker tests. However, each of methods have their own limitations. Colonoscopy is the gold standard diagnostic test for CRC, but it is invasive, and resources are limited in some healthcare systems, making it a poor candidate for population screening. Less invasive methods, such as stool‐based or blood‐based tests, are preferable screening techniques, but current methods lack in diagnostic accuracy. For example, the faecal immunochemical test (FIT) is a non‐invasive test that detects for traces of blood in a stool sample, but the diagnostic performance is limited, particularly for early‐stage CRC cancers and pre‐cancerous conditions. In a systematic‐review by Monahan *et al.*, sensitivity of FIT for CRC has been reported varying from 64.1% to 93.4% (with specificity from 76.9% to 95.0%) across 23 cohort studies published between May 2018 and November 2020, including 69 536 symptomatic adults from primary care.^29^ In their latest study, Imperiale *et al.* have reported a FIT sensitivity of 67.3% (with specificity of 94.8%) for all stages of CRC combined; however, FIT sensitivity dropped to 50% for Stage I CRC.^30^ There are currently two blood‐based tests employed for CRC screening; one of them only recently FDA approved.^31^ Various liquid biopsies are still under development.

Additional difficulties contributing to the delayed diagnosis, and consequently increased incidence of advanced‐stage disease in CRC patients, can be lower disease awareness, lack of screening, underappreciation of symptoms and reluctance to seek medical care due to fear and/or negative perception around stool specimen collection and colonoscopy.^19^ This highlights the importance of understanding the risk factors that are at play, including patient lifestyle factors and environmental exposures. By integrating these diverse data sources, we can develop more effective screening strategies and diagnostic tools that can identify cancer at an earlier, more treatable stage.

### 3.3 Metabolic and immune responses in the pre‐cancerous state

**FIGURE 2:**
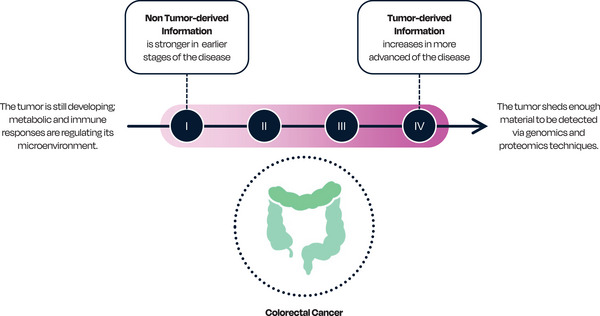
Colorectal cancer stage development and key diagnostic information. Non tumour‐derived and tumour‐derived information are shed in different quantities throughout cancer development. Clinical performance evaluations suggest that current genomics and proteomics techniques do not sufficiently detect non tumour‐derived information in preliminary stages of the disease.

As a pathological and molecularly heterogeneous cancer, CRC is influenced by both exogenous and endogenous factors.^32^ The spectrum of molecular characteristics of CRC across different bowel sub‐sites highlights the interactive roles of metabolism and the immune system, providing what is defined as ‘non tumour‐derived information’ (Figure 2), which are key to comprehend the steps leading to tumorigenesis.^32^, ^33^

The reprogramming of cell metabolism—an established hallmark of cancer—occurs in both malignant and premalignant stages of CRC, suggesting its pivotal role in the progression of CRC carcinogenesis.^33^, ^34^, ^35^ Similarly, the inflammatory state of premalignant lesions driven by immune cells, enables both cancer formation and progression.^33^ This can primarily be evidenced by colitis‐associated carcinogenesis (CAC), where chronic inflammation can lead to the formation of colonic carcinogenesis. Hardbower *et al.* showed the profound effect of EGFR‐signalling in macrophages on the development of CAC.^36^ On the other hand, polymorphonuclear neutrophils can increase DNA double‐strand break burden and promote genomic instability—another hallmark of cancer.^33^, ^37^

Polymorphic microbiomes also play a role in CRC pathogenesis. Research suggests that in genetically predisposed FAP patients, intestinal microbial dysbiosis and the loss of T cells (i.e., immune cells) impair mucosal immunity and tumour surveillance.^38^ Bacterial chronic infections and cigarette smoke can also be risk factors for CRC formation and progression by affecting the gut microbiota and their metabolites, which influence oncogenic signalling pathways.^39^, ^40^

## 4 CURRENT COLORECTAL CANCER DIAGNOSTIC TESTING

### 4.1 Screening programmes

**FIGURE 3:**
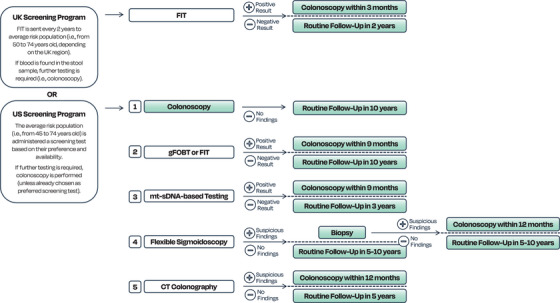
Current colorectal cancer screening options in the UK and US with testing and follow‐up timelines. Their workflow is self‐explanatory through figure related text. [FIT: faecal immunochemical test; gFOBT: guaiac‐based faecal occult blood test; mt‐sDNA: multitargeted stool DNA; CT: computed tomography].

CRC screening programmes were introduced around 20 years ago worldwide and are now widely employed in an attempt to identify CRC at early stages of the disease, before symptoms appear.^41^ The current screening pathway in the UK involves a preliminary non‐invasive faecal test (e.g., FIT; Figure 3).^42^ If abnormalities are detected in the preliminary tests, these are followed by colonoscopy, which might also include a tissue biopsy to assess the status of any findings (e.g., adenomatous polyps). Tissue biopsies are examined by pathologists to search for cancerous cells and can undergo further molecular tests for specific genetic mutations depending on the tumour biology. KRAS, NRAS and BRAF genes are often investigated, as well as proteomic changes in the production of tumour suppression proteins (e.g., HER2); more frequently MSI or MMR genes changes are targeted to aid in therapeutic management.^43^ In the USA, the average risk population has access to various screening options, which also include stool‐based testing and colonoscopy (Figure 3).^44^

Access to screening differs depending on the governing healthcare system. In the UK, people can receive a FIT screening kit from the age of 50 years old in Scotland, 51 in Wales and 60 in England and Northern Ireland, up to 74 years old.^42^ In the US, only people with health insurance can access screening programmes. The Centers for Medicare & Medicaid Services has recently lowered the minimum age for coverage for certain CRC screening tests to 45 years old, such as flexible sigmoidoscopy, guaiac‐based faecal occult blood test (gFOBT), FIT, multi‐targeted stool DNA (mt‐sDNA) based test and blood‐based biomarker tests.^45^ They have stated that for a blood‐based biomarker test to meet the criteria for coverage, the test must be able to achieve CRC sensitivity ≥ 74% and specificity ≥ 90%.^46^

The decision to lower the minimum age for coverage of CRC screening has been praised for addressing the great burden of early‐onset CRC in younger people aged 45‐49. However, the incidence of CRC in younger people is still lower than in older people, hence prevalence and positive predictive values will be diminished, leading to significant costs associated with reducing the screening age, which could negatively impact the funds allocated to those at higher risk, due to socioeconomic or environmental factors. Furthermore, the possibility of over‐burdened healthcare systems and longer waiting times is a consideration;^21^ the cost of a novel liquid biopsy test for the younger population will need to take into account these factors and be optimized for cost‐effectiveness.

### 4.2 New tests for colorectal cancer detection

Until last year, only two U.S. FDA approved diagnostic technologies were available as an alternative to FIT: Cologuard™ (Exact Science) and Epi proColon™ ((Epigenomics AG), also known as ColoVantage™ and commercialized by Quest Diagnostics). However, the FDA approved three additional tests in 2024. ColoSense™ (Geneoscopy) and Shield™ (Guardant Health) were approved in May and July 2024 respectively;^31^, ^47^ and Cologuard Plus™ (Exact Science) in October 2024.^48^ Their clinical performance metrics are shown in Table 1 along with FIT performance,^30^, ^49^, ^50^, ^51^, ^52^ and costs whether available.

**TABLE 1:** Current tests for colorectal cancer (CRC) detection.

Name	Company	Test category	Technology	Specificity for CRC % (*n* total cases)	Sensitivity for CRC % (*n* total cases)	Sensitivity for CRC Stage I % (*n* total cases)	Sensitivity for AA % (*n* total cases)	Study/Trial	Cost
FIT	N/A	Stool‐based	Haemoglobin detection assay	95 (17 934)	67 (98)	50 (30)	23 (2144)	BLUE‐C^30^	Available under $100
Cologuard	Exact Sciences	Stool‐based	mt‐sDNA assay	90 (4457)	92 (65)	90 (29)	42 (757)	DeeP‐C^49^	$500
^*^Cologuard Plus	Exact Sciences	Stool‐based	mt‐sDNA assay	91 (17 934)	94 (98)	87 (30)	43 (2144)	BLUE‐C^30^	$790
^*^ColoSense	Geneoscopy	Stool‐based	mt‐sRNA assay	88 (3760)	94 (36)	100 (14)	46 (606)	CRC‐PREVENT^50^	Product not yet available
Epi proColon (ColoVantage)	Epigenomics AG (Quest Diagnostics)	Blood‐based	Plasma methylated SEPT9 DNA assay	79 (444)	68 (44)	41 (17)	22 (621)	PRESEPT^51^	$800
^*^Shield	Guardant Health	Blood‐based	Plasma cell‐free DNA assay	90 (6680)	83 (65)	65 (17)	13 (1116)	ECLIPSE^52^	$1,500

* Recently approved by the FDA in 2024.^31^, ^47^, ^48^ ^.^

Other liquid biopsies can also be used when CRC is suspected. Carcinoembryonic antigen (CEA) is a common biomarker in blood that is investigated for colorectal abnormalities. However, CEA is not specific for CRC; it can indicate the presence of several cancers when elevated, and it is mainly indicated for prognosis and treatment management.^53^

Most screening methods on the market for the detection of CRC still lack sensitivity for early‐stage cancer. The FDA approval of newer tests with higher sensitivity for stage I CRC represents a step forward for early diagnostics.^31^, ^47^, ^50^, ^52^ One noticeable limitation of stool‐based tests is the lack of adherence. A study with over 10 000 participants reported that less than 25% complied with FIT testing in a 12–15 month follow‐up screening window, compared to the recently assessed 96% adherence of a blood‐based liquid biopsy during validation studies.^54^, ^55^ There is also a need for an alternative to current genetic biomarker panels, which could be represented by a new range of blood‐based liquid biopsies that avoid focussing on selected genetic markers (e.g., DNA methylation) or circulating tumour cells (CTCs), and explore a wider range of biological information contained in blood which can look at the overall disease signature (i.e., tumour‐ and non‐tumour‐derived information). Blood‐based genetic technologies tend to have high specificity, which is beneficial for controlling overdiagnosis, but these tests often have low sensitivities, particularly for smaller or early‐stage tumours.^33^, ^56^ Unfortunately, this means many early‐stage CRC tumours will be missed.

Whether blood‐based liquid biopsies can fully replace stool‐based tests, or be offered as an alternative, remains to be seen. Technologies are striving to improve their performance through the combination of results with clinical markers, such as metabolomics and proteomics biomarkers, and specific risk factors (e.g., age, diet, concurrent diseases).^33^, ^56^

## 5 PROSPECTIVE DIAGNOSTICS FOR COLORECTAL CANCER

Liquid biopsy research has attracted vast interest in recent years, particularly in the field of CRC diagnostics.^57^, ^58^ Freenome has also recently announced the results of their pivotal clinical study. In the PREEMPT CRC^®^ validation study, Freenome's CRC blood test obtained 79.2% sensitivity for all CRC stages and 57.1% for Stage I CRC (91.5% specificity for non‐advanced colorectal neoplasia).^59^, ^60^ Table 2 overviews also two other emerging diagnostic tests for CRC along with their reported performance^60^, ^61^, ^62^ and costs whether available; EarlyTect^®^‐C (stool‐based) by Genomictree and ColoxNGS (blood‐based) by Novigenix.

**TABLE 2:** Emerging diagnostic tests for colorectal cancer (CRC) detection.

Name	Company	Test category	Technology	Specificity for CRC % (*n* total cases)	Sensitivity for CRC % (*n* total cases)	Sensitivity for CRC Stage I % (*n* total cases)	Sensitivity for AA % (*n* total cases)	Study/Trial	Cost
EarlyTect^®^‐C	Genomictree	Stool‐based	Plasma methylated SDC2 DNA assay	90 (245)	90 (245)	85 (55)	67 (3)	NEXT CRC^61^	Price not publicly disclosed
Freenome CRC Test	Freenome	Blood‐based	Plasma cell‐free DNA assay	92 (24 567)	79 (72)	57 (28)	13 (2567)	PREEMPT CRC^59^, ^60^	Price not publicly disclosed
ColoxNGS	Novigenix	Blood‐based	29‐gene panel and plasma biomarkers assay	90 (149)	80 (97)	67 (44)‡	55 (103)	DGNP‐COL‐0310^62^	$350 (CHF 279)

^‡^ ColoxNGS results include both CRC stage I and II.

### 5.1 Prospective proteomics for CRC

The application of protein screening technologies to identify diagnostic cancer biomarkers has also seen a significant increase, owing to industry driven standardisation and application to a range of large‐scale biobanking facilities.

Proteins and their derivatives are increasingly recognised as promising biomarkers for the early detection of cancer. Their structural complexity and dynamic changes during tumorigenesis offer valuable insights into disease‐specific molecular alterations. Glycoproteins (i.e., proteins with carbohydrate chains) are involved in key physiological processes including cell signalling, immune modulation and maintenance of tissue architecture. Aberrant glycosylation is a hallmark of malignant transformation and occurs early in CRC progression, making glycoproteins attractive candidates for non‐invasive biomarker development.^63^ Takakura *et al.* applied a targeted O‐glycoproteomics approach to serum samples and identified 2068 glycoforms, with 44 specifically associated with advanced CRC.^64^ Furthermore, lipoproteins (i.e., complexes of proteins and lipids) have gained attention as potential biomarkers in CRC; Xu *et al.* reported that decreased levels of lipoproteins were associated with CRC, yielding 85.7% sensitivity and 93.3% specificity when investigating differences in levels of apolipoprotein A2 between cancerous and healthy patients.

Proteomic technologies—such as Olink^®^, SomaScan^®^ and the Seer^®^ platforms—have yet to prove their utility in large validation studies for CRC; however, the advancements in the field and optimisation in current workflows to achieve high‐throughput analysis could also boost cost‐effectiveness, laying the foundation for their use in fast‐paced clinical settings.^65^

#### 5.1.1 Olink^®^ Platform

**FIGURE 4:**
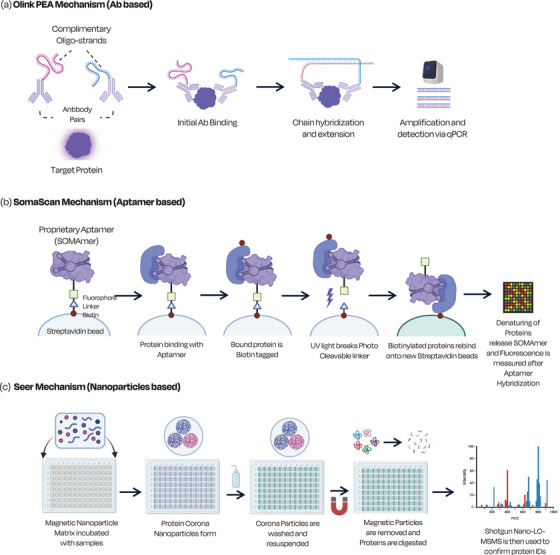
Mechanistic workflows of prospective proteomics for CRC diagnostics. Olink (A), SomaScan (B) and Seer (C) analytic platforms select and identify proteins; their workflow is self‐explanatory through figure related text. [PEA, proximity extension assay; qPCR, quantitative polymerase chain reaction; LC‐MSMS, liquid chromatography tandem mass spectrometry].

The Olink platform is an oligonucleotide amplification‐based approach where paired antibodies are used to complex with a protein target of interest (Figure 4A). Amplification and detection via quantitative polymerase chain reaction (qPCR) allow for identification and quantification of selected proteins (e.g., biomarkers). The technology has already been used to measure approximately 3000 blood derived proteins samples from 54 000 participants of the UK Biobank.^66^ Bhardwaj *et al.* have explored the potential of plasma proteins for early detection of CRC.^67^ In specific, they used Olink's proximity extension assay (PEA) for measurement of five blood protein markers, including amphiregulin, and obtained an area under the curve (AUC) of 0.82 for all CRC stages (*n* = 56), 0.86 for early stage CRC (*n* = 23) and 0.60 for AA (*n* = 101), showing promising results to be tested in larger clinical validation studies. Qian *et al.* investigated a panel of five inflammatory proteins for CRC detection, obtaining an AUC of 0.80 in their validation set; however, a lower AUC of 0.59 was calculated from their diagnostic performance validation set (true screening setting), showing limitations of reproducibility in a real clinical scenario.^68^

#### 5.1.2 SomaScan^®^ Platform

The SomaScan platform is based onto proprietary aptamers use. Their aptamers, which are molecular binders, are defined as SOMAmers and consist of single stranded oligonucleotides that fold into defined architectures and can be tuned towards selected proteins (Figure 4B). Through binding, cleavage and subsequent rebinding, the selected protein denatures and releases the SOMAmer, which produces a measurable fluorescent signal. The SOMA (Space Omics and Medicine Atlas) technology has been compared to Olink in both the UK Biobank and the INTERVAL study to develop multiomics datasets that correlate to specific genetic scores. ^66^, ^69^ However, these datasets can only be built with projects that have gene sequencing data collected. Li *et al.* have investigated SOMAmers of 1317 proteins to screen and validate novel stool protein biomarkers of CRC; 92 proteins were found significantly elevated in CRC samples (*n* = 76) compared to healthy controls (*n* = 63).^70^

#### 5.1.3 Seer^®^ Proteograph^™^ Platform

The Seer platform uses a magnetic nano‐particle approach that focusses on analysing the deep proteome by preselecting ultra‐low level abundance proteins. A magnetic nano‐particle mixture is added to a plate containing serum or plasma and, depending upon the structure of the magnetic particle, a specific protein complex to form a unique protein corona (Figure 4C). A step‐by‐step process leads to the production of a peptide mixture appropriate for Shotgun proteomics to identify the proteins of interest. The Seer approach has already shown its detection ability and has been applied to proteogenomic landscaping in breast cancer.^71^ In June 2025, a press release from Seer has announced a partnership with Korea University for a large‐scale 20 000‐sample proteomics study to develop AI‐driven cancer diagnostics; the study aim is to obtain an early detection tool that is more sensitive, scalable and patient‐personalized.^72^

### 5.2 The importance of non tumour‐derived information

Metabolic and immune system mechanisms are key factors that have been investigated to comprehend the signals to examine non tumour‐derived information in order to achieve earlier cancer detection. However, the specific biomarkers that can determine premalignant and earlier stages of cancer have yet to be identified.^33^ Recent studies in proteomics and metabolomics found that certain plasma proteins and metabolites are highly associated with increased CRC susceptibility, presenting scope for further investigation in both proteomics and metabolomics non‐tumour biomarkers for earlier cancer detection.^73^, ^74^, ^75^, ^76^, ^77^

Diagnostic tools that detect chronic inflammation driven by immune cells, microbial imbalance in the gut microbiome also affecting the metabolism, and hereditary cancer syndromes could inform risk assessment, early detection, and personalized treatment plans.^36^, ^78^ Further research focussed onto understanding non‐tumour‐derived information and developing technologies able to specifically identify and target these signals can be key to achieve early detection of cancer.

### 5.3 Multi‐omic spectral analysis

There are various other analytical techniques that do not naturally fit under the single ‘‐omic’ umbrella, such as infrared (IR) spectroscopy. The interest in IR spectroscopy for biomedical applications has gradually increased due to the need for tests that are robust, affordable, easy‐to‐use and minimally invasive. There are a plethora of proof‐of‐concept studies that have demonstrated the effectiveness of the technique for the analysis of biofluids and tissue specimens.^79^, ^80^ IR spectroscopy does not require extensive sample preparation such as isolation and extraction of DNA, contributing to cost‐ and time‐effectiveness.^33^, ^81^

#### 5.3.1 Infrared spectroscopy

IR spectroscopy is based on the interaction between IR light and the molecules contained in biological and chemical compounds. When IR light interacts with these molecules, their bonds vibrate at different but specific wavelengths. The vibrations are based on the atoms and bonds in the sample's molecules. For example, proteins are formed by amino acids, which are connected through amide groups known as peptide bonds; these are structurally different from molecular bonds in carbohydrates, and therefore these two macro‐compounds vibrate at different wavelengths in the IR spectral range.^33^

**FIGURE 5:**
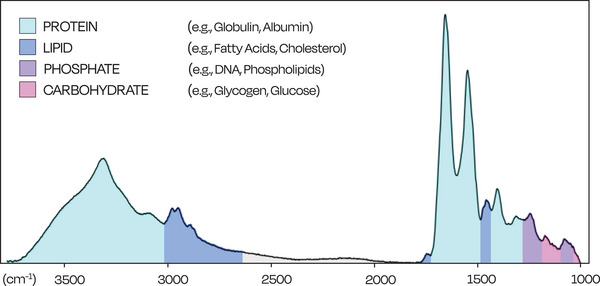
Infrared spectrum detailing the main components of blood serum. Human blood serum spectral signature contains the information regarding proteins (e.g., amino acids), lipids (e.g., cholesterol), phosphates (e.g., DNA) and carbohydrates (e.g., glucose) present in a blood sample.

The spectrum of a biological fluid, such as human blood serum, can be seen in Figure 5. The subtle differences disease classes within the spectral data is detected by machine learning algorithms, allowing classification of a wide range of cancers.^41^, ^42^

An IR spectroscopy‐based liquid biopsy for cancer detection (Dxcover Ltd.) has shown promise in recent years.^82^ The development of the technology started with several proof‐of‐concept studies for various cancer types, more recently progressing into large‐scale prospective studies.^83^, ^84^, ^85^

In a multi‐cancer study, human blood serum of 200 CRC patients was analysed against serum from 459 patients which were suspected of having cancer, but ultimately had a negative diagnosis (i.e., no presence of malignancy).^85^ This discovery study reported the ability of the algorithm to detect 100% of Stage I and II adenocarcinomas, with overall 91% sensitivity and 76% specificity. When the machine learning model was tuned for greater specificity, the algorithm reported 77% sensitivity for CRC detection at 90% specificity, which surpasses the minimum requirements of the Centers for Medicare & Medicaid Services guidelines.^85^ This technology is still under development and currently going through clinical validation studies.

## 6 CONCLUSIONS

CRC is becoming a disturbing healthcare burden with the number of cases likely to increase steadily over the next decade, with growing concern over the rise in early‐onset cases.^1^ Diagnosing CRC in its earlier stages is imperative to improve prognosis and survival rates, while reducing the cost of care and treatment of patients. Worldwide, most CRC screening pathways target people aged over 50, and the USA only recently reduced the recommended screening age to 45.^45^, ^46^ This means that many individuals in the younger population do not have the option to test for CRC, and early‐onset cases can be easily missed. In addition, colonoscopy—the gold standard for CRC diagnosis—is often not accessible in many healthcare systems for average‐risk screening. A cost‐effective, fast and less invasive screening option would be beneficial to streamline those most at risk for a colonoscopy.

Emerging diagnostics aim to increase the accuracy of current detection tools (e.g., FIT), obtaining high sensitivity with a set ≥ 90% specificity. Blood‐based liquid biopsies from leading companies reported validation studies with impressive results, yet lack in sensitivity for early‐stage cancer and pre‐cancer. These technologies employ genomics and proteomics assays with high sensitivity for targeted genes/proteins, which are generally present only at later stages of tumorigenesis. Some stool‐based tests (e.g., Cologuard) boast a higher sensitivity for early‐stage CRC, but stool‐based tests reportedly have low adherence compared to blood‐based liquid biopsies.^54^, ^55^

A better understanding of tumour progression from premalignant stages into CRC and the related changes in cancer biology are vital for the future of CRC diagnostics. Multi‐omic tests that examine various phenomena, rather than solely focussing on one single‐omics, such as solely genomics, are likely to be at the forefront of liquid biopsy research. Innovative technologies that assess signals related to the tumour and the non‐tumour environment may pave the way for the earlier detection of CRC.

## AUTHOR CONTRIBUTIONS

**Alexandra Sala**: Conception, design, drafting and reviewing. **Lisa van den Driest**: Drafting and reviewing. **Nicholas J. W. Rattray**: Conception, design, drafting and reviewing. **Caroline H. Johnson**: Drafting and reviewing. **James M. Cameron**: Design, drafting and reviewing. **Sajid A. Khan**: Reviewing. **David S. Palmer**: Reviewing. **Matthew J. Baker**: Conception and reviewing. All authors read and approved the final manuscript.

## FUNDING INFORMATION

All funding has been allocated from Dxcover Ltd.

## CONFLICT OF INTEREST STATEMENT

Alexandra Sala and James M. Cameron are the employees of Dxcover Ltd. David S. Palmer and Matthew J. Baker are the founders and directors of Dxcover Ltd.

## CONSENT FOR PUBLICATION

Not applicable.

## ETHICS STATEMENT

The authors have nothing to report.

## Data Availability

Not applicable.
